# Risk factors for ocular surface squamous neoplasia in Kenya: a case–control study

**DOI:** 10.1111/tmi.12792

**Published:** 2016-10-24

**Authors:** Stephen Gichuhi, Ephantus Macharia, Joy Kabiru, Alain M'bongo Zindamoyen, Hillary Rono, Ernest Ollando, Joseph Wachira, Rhoda Munene, Timothy Onyuma, Walter G. Jaoko, Mandeep S. Sagoo, Helen A. Weiss, Matthew J. Burton

**Affiliations:** ^1^ International Centre for Eye Health London School of Hygiene and Tropical Medicine London UK; ^2^ Department of Ophthalmology University of Nairobi Nairobi Kenya; ^3^ PCEA Kikuyu Eye Unit Kikuyu Kenya; ^4^ Kitale District Hospital Kitale Kenya; ^5^ Sabatia Eye Hospital Wodanga Kenya; ^6^ Kenyatta National Hospital Nairobi Kenya; ^7^ Department of Pathology MP Shah Hospital Nairobi Kenya; ^8^ KAVI Institute of Clinical Research University of Nairobi Nairobi Kenya; ^9^ UCL Institute of Ophthalmology London UK; ^10^ Moorfields Eye Hospital London UK; ^11^ St. Bartholomew's Hospital London UK; ^12^ MRC Tropical Epidemiology Group London School of Hygiene and Tropical Medicine London UK

**Keywords:** ocular surface squamous neoplasia, risk factors, ultraviolet radiation, HIV, antiretroviral therapy, allergic conjunctivitis, néoplasie squameuse de la surface oculaire, facteurs de risque, rayonnement ultraviolet, VIH, traitement antirétroviral, conjonctivite allergique

## Abstract

**Objective:**

To determine modifiable risk factors of ocular surface squamous neoplasia (OSSN) in Kenya using disease‐free controls.

**Methods:**

Adults with conjunctival lesions were recruited at four eye care centres in Kenya and underwent excision biopsy. An equal number of controls having surgery for conditions not affecting the conjunctiva and unrelated to ultraviolet light were group‐matched to cases by age group, sex and eye care centre. Associations of risk factors with OSSN were evaluated using multivariable logistic regression to estimate odds ratios (ORs) and 95% confidence intervals (95% CIs). Continuous variables were compared using the t‐test or the Wilcoxon–Mann–Whitney *U*‐test depending on their distribution.

**Results:**

A total of 131 cases and 131 controls were recruited. About two‐thirds of participants were female, and the mean age of cases and controls was 42.1 years and 43.3 years, respectively. Risk factors for OSSN were HIV infection without antiretroviral therapy (ART) use (OR = 48.42; 95% CI: 7.73–303.31) and with ART use (OR = 19.16; 95% CI: 6.60–55.57), longer duration of exposure to the sun in the main occupation (6.9 h/day *vs*. 4.6 h/day, OR = 1.24; 95% CI: 1.10–1.40) and a history of allergic conjunctivitis (OR = 74.61; 95% CI: 8.08–688.91). Wearing hats was protective (OR = 0.22; 95% CI: 0.07–0.63).

**Conclusion:**

Measures to prevent and control HIV, reduce sun exposure such as wearing hats and control allergic conjunctivitis are recommended.

## Abstract (fr)

**Objectif:**

Déterminer les facteurs de risque modifiables de néoplasie squameuse de la surface oculaire (NSSO) au Kenya en utilisant des témoins sans la maladie.

**Méthodes:**

Des adultes atteints de lésions conjonctivales ont été recrutés dans quatre centres ophtalmiques au Kenya et ont subi une biopsie par excision. Un nombre égal de témoins ayant subi une intervention chirurgicale pour des affections n'affectant pas la conjonctive et sans aucun lien avec la lumière ultraviolette ont été appariés aux cas par groupe, selon l’âge, le sexe et le centre de soins oculaires. Les associations entre les facteurs de risque et la NSSO ont été évaluées à l'aide de la régression logistique multivariée pour estimer les odds ratios (OR) et les intervalles de confiance à 95% (IC95%). Les variables continues ont été comparées en utilisant le test t ou le test U de Wilcoxon‐Mann‐Whitney, selon leur distribution.

**Résultats:**

Un total de 131 cas et 131 témoins ont été recrutés. Environ deux tiers des participants étaient des femmes et l’âge moyen des cas et des témoins était respectivement de 42,1 et 43,3 ans. Les facteurs de risque pour la NSSO étaient l'infection par le VIH sans traitement antirétroviral (OR = 48,42; IC95%: 7,73‐303,31) et avec traitement ART (OR = 19,16; IC95%: 6,60‐55,57), une plus longue exposition au soleil dans l'occupation principale (6,9 heures/jour vs 4,6 heures/jour, OR = 1,24; IC95%: 1,10‐1,40) et des antécédents de conjonctivite allergique (OR = 74,61; IC95%: 8,08‐688,91). Le port de chapeau était protecteur (OR = 0,22; IC95%: 0,07‐0,63).

**Conclusion:**

Des mesures de prévention et de lutte contre le VIH, la réduction de l'exposition au soleil avec par exemple le port de chapeau et la lutte contre la conjonctivite allergique sont recommandées.

## Abstract (es)

**Objetivo:**

Determinar los factores de riesgo modificables de neoplasia escamosa de la superficie ocular (NESO), en Kenia y utilizando controles sanos.

**Métodos:**

Se reclutaron adultos con lesiones en la conjuntiva en cuatro centros oftalmológicos de Kenia, y se les realizó una biopsia por escisión. Los casos se parearon por edad, sexo y centro oftalmológico con un número igual de controles, pacientes que se habían sometido a cirugía por otras condiciones que no afectaban la conjuntiva y que no estaban relacionadas con luz ultravioleta. Se evaluaron las asociaciones a factores de riesgo con NESO mediante regresión logística multivariable para calcular en cada caso la razón de probabilidades (odds ratio ‐ OR) y los intervalos de confianza (IC) del 95%. Las variables continuas se compararon utilizando las pruebas de T o de Wilcoxon‐Mann‐Whitney U, dependiendo de su distribución.

**Resultados:**

Se reclutaron en total 131 casos y 131 controles. Aproximadamente un tercio de los participantes eran mujeres y la edad media de los casos y controles era de 42.1 años y 43.3 años respectivamente. Los factores de riesgo para NESO eran tener infección por VIH sin estar recibiendo tratamiento retroviral (TAR) (OR=48.42; IC 95% 7.73‐303.31) y recibiendo TAR (OR=19.16; IC 95% 6.60‐55.57), una mayor duración de la exposición al sol en su principal ocupación (6.9 hrs/día vs 4.6 hrs/día, OR=1.24; IC 95% 1.10‐1.40) y una historia de conjuntivitis alérgica (OR=74.61; 95%CI 8.08‐688.91). El utilizar sombrero era un factor de protección (OR=0.22; IC 95% 0.07‐0.63).

**Conclusión:**

Se recomienda la implantación de medidas para prevenir y controlar el VIH, las que reducen la exposición al sol ‐ como por ejemplo el utilizar sombreros ‐ y el control de la conjuntivitis alérgica.

## Introduction

Ocular surface squamous neoplasia (OSSN) is a spectrum of disease in the conjunctiva and cornea that ranges from intra‐epithelial neoplasia to invasive squamous cell carcinoma. It usually presents as a unilateral tumour on the eye, centred around the corneal limbus (the junction between the cornea and conjunctiva) 1.

The epidemiology, aetiology and pathophysiology of OSSN are not well understood. There appear to be two disease patterns: a milder form in temperate climates and a more aggressive one in sub‐Saharan Africa (SSA). In SSA, both males and females have equal incidence rates, although in prevalence series females constitute 60–70%, in contrast to other regions where males predominate 2, 3. It is unclear why the prevalence is not 1:1 in African series. The presentation of OSSN in SSA also is relatively more aggressive, with a short history of 3–8 months compared to one year in temperate climates 4, 5, 6, 7, and younger age of onset (30–40 years in SSA *vs*. 60–70 years in temperate climates) 2, 3, 8. Current evidence suggests that multiple factors may combine to drive the disease process. Case–control studies and cancer registry data implicate ambient solar ultraviolet radiation (UVR) among other factors. The risk of OSSN is higher in those who spend more time outdoors and the incidence worldwide varies with latitude peaking at 16°S, particularly in the sunny areas of Eastern and Southern Africa, and also Australia 2, 9, 10, 11. This may be related to the Earth's 23° tilt and elliptical orbit which brings it closer to the sun during the southern summers (perihelion) than during the northern summer (aphelion) 12. The *TP‐53* gene mutation, which is associated with UV radiation, has been found more frequently in OSSN tissue 11, 13.

There is a strong association between OSSN and HIV in SSA, and incidence rose sharply with the onset of the HIV pandemic 14. However, about 30% of people with OSSN in SSA are not infected with HIV, suggesting that other factors also contribute 2. The association between human papilloma virus (HPV) and OSSN is inconclusive 15, 16, 17, 18, 19, 20. This is probably because of variations in methodology and the specific HPV types that have been looked for 2.

The importance of vitamin A in maintaining the health of the ocular surface is established, and its deficiency leads to goblet cell loss, desquamation and keratinisation of the ocular surface 21. Vitamin A deficiency (serum retinol <30 μg/dl or <1.05 μmol/l) is common in HIV patients 22. The potential role of vitamin A deficiency in OSSN has not previously been investigated. Cigarette smoking, although associated with many cancers, has not been conclusively shown to be associated with OSSN 2.

To date, case–control studies investigating risk factors for OSSN have enrolled individuals with conjunctival lesions suspected to be OSSN, which were excised and sent for histopathology. Individuals with OSSN were classified as ‘cases’ and those with benign lesions as ‘controls’ 6, 11, 13, 23. However, these are probably not appropriate controls, as benign conjunctival lesions may have some risk factors in common with OSSN. For example, pterygia, actinic keratosis and papillomas, the most common benign lesions, may also be associated with solar UV radiation, p53 gene mutation and human papillomavirus infection 24, 25, 26, 27.

The aim of this study was to investigate the role of multiple risk factors (HIV infection, vitamin A, cigarette smoking, ultraviolet light exposure and occupation), which are preventable or modifiable, by comparing OSSN cases to disease‐free controls presenting at four eye care centres in Kenya.

## Methods

### Ethical approval

This study was approved by the Kenyatta National Hospital – University of Nairobi Ethics and Research Committee and the London School of Hygiene & Tropical Medicine Ethics Committee. All participants gave informed written consent to take part. It adhered to the tenets of the Declaration of Helsinki.

### Study design

A frequency‐matched case–control study design was used, with a 1:1 case‐to‐control ratio. Matching was by age (10‐year age groups) and sex, as both are strong constitutional factors associated with OSSN. Cases of OSSN (subsequently confirmed by histology to have OSSN) were compared with controls who had no conjunctival lesion, attending for routine ophthalmic surgery that involved incision of the conjunctiva. Controls were recruited at the same ophthalmic clinics after cases were enrolled.

### Study setting

Recruitment was between July 2012 and July 2014 in four eye care centres in Kenya, namely Kenyatta National Hospital (KNH) Eye Clinic, Nairobi; PCEA Kikuyu Eye Unit (KEU), Kikuyu near Nairobi; Sabatia Eye Hospital (SEH), Vihiga about 350 km north‐west of Nairobi near Lake Victoria; and Kitale District Hospital (KDH) in the North Rift Valley. These are busy eye units that receive referrals from surrounding areas (Table 1).

**Table 1:** Study Centres and the 2013 HIV prevalence in their catchment areas 46

Study centre	County^b^	HIV prevalence (%)
Kenyatta National Referral Hospital^a^	Nairobi	8.0
	Machakos	5.0
	Kajiado	4.4
PCEA Kikuyu Hospital	Kiambu	3.8
	Muranga	5.2
	Nyeri	4.3
	Embu	3.7
	Kirinyaga	3.3
Sabatia Eye Hospital	Vihiga	3.8
	Kakamega	5.9
	Bungoma	3.2
	Kisumu	19.3
	Homa Bay	25.7
	Siaya	23.7
	Migori	14.7
Kitale District Hospital	Trans Nzoia	5.1
	Turkana	7.6
	West Pokot	2.8

The prevalence data refer to adults of age 15–49 years. The national prevalence was 6.0%.

a The national referral hospital receives patients from further afield including counties that may be listed under other study centres.

b The first county listed is where the study centre is located.

### Study population, inclusion and exclusion criteria

Both cases and controls were recruited from people attending the eye clinic. Adult patients (aged >18 years) with any histologically confirmed OSSN tumour (first presentation or recurrence) were included as cases. Controls were adult patients scheduled for ocular surgery that involved a conjunctival incision for a disease not involving the conjunctiva and unrelated to ultraviolet light (such as trauma, squint or retinal surgery). History of prior surgery in the index eye was an exclusion criterion for controls. Eligible participants who gave written informed consent to participate were enrolled.

### Demographic and occupational data

Data on age, sex, marital status and educational history were collected. Questions included the following: residential history (regions lived in for >1 year), lifetime occupational history (including whether based mainly indoors or outdoors), hours of sun exposure on an average day, patterns of wearing hats, caps or sunglasses, and cigarette smoking history. Participants with regular partners who smoked cigarettes were classified as passive smokers.

### Clinical examination

For potential cases (participants with conjunctival lesions), a detailed clinical and slit lamp examination was performed, to document clinical features of the tumours. High‐resolution digital photographs were taken. For potential controls, the ocular surface was examined at the slit lamp before surgery to exclude any conjunctival lesions.

### Surgical excision and histopathology

All lesions were excised under local infiltration anaesthetic using an operating microscope with a 4‐mm clear margin, using the ‘no touch technique’ to minimise tumour handling. Specimens were placed directly into buffered formalin and subsequently examined at the histopathology laboratory at the MP Shah Hospital, Nairobi. All the histology slides were stained with haematoxylin and eosin and examined by one pathologist (TO). For controls, a 2‐mm‐by‐2‐mm piece of conjunctiva was excised and immediately placed in RNAlater tissue collection RNA stabilisation solution (Ambion Inc., Austin, Texas, USA) for further molecular studies, which will be reported separately.

### HIV/CD4 count assays

Individuals with a confirmed diagnosis of OSSN were tested for HIV and CD4 cell count 4 weeks after the surgery when they returned for results. Controls were tested for HIV and CD4 at the time of surgery. Pre‐test counselling was provided individually to all participants by a counsellor. Venous whole blood was tested at the Kenya Aids Vaccine Institute (KAVI) for Clinical Research Laboratory at the University of Nairobi for HIV initially using Vironostika antigen/antibody kit (Biomerieux, Marcy l'Etoile, France) then later the Alere Determine HIV‐1/2 Ag/Ab (Alere, CITY, USA) and Trinity Unigold (Trinity Biotech, Jamestown, USA) when Vironostika became unavailable. CD4 count was measured using FacsCount (Becton Dickinson, Franklin Lakes, USA). Samples from Sabatia Eye Hospital and Kitale District Hospital were tested for HIV using Alere Determine HIV‐1/2 Ag/Ab (Alere, CITY, USA) and for CD4 count using FacsCount (Becton Dickinson, Franklin Lakes, USA). We also asked about antiretroviral (ART) use.

### Serum vitamin A assays

Blood samples collected without EDTA were immediately wrapped in aluminium foil to prevent degradation from light. They were transported to the KAVI laboratory in a cool box within an hour from nearby centres (KNH and KEU) or couriered on ice packs from more distant centres (SEH and KDH). On arrival, samples were immediately centrifuged at 2500 revolutions per minute for 10 min in a dimly lit room, aliquoted into cryovials, labelled, rewrapped in aluminium foil and stored in a freezer at −80 °C. Samples were batched and shipped on dry ice to the Tropical Diseases Research Centre's Nutrition Unit in Ndola, Zambia. Retinol was quantified using high‐performance liquid chromatography 28. The quantitation of retinol was performed using the SHIMADZU Prominence HCT2010 HPLC (Kyoto, Japan). Individuals with serum retinol <30 μg/dl were considered vitamin A deficient.

### Sample size determination

This case–control study was nested within a randomised controlled trial of post‐operative 5‐fluorouracil eyedrops to reduce recurrent OSSN. The sample size of 131 cases and 131 controls provides 90% power to detect a difference in prevalence of a given exposure of 40% in controls and 60% in cases.

### Statistical analysis

Data were managed in an Access database (Microsoft), cleaned and transferred into STATA version 12.1 (StataCorp, College Station, Texas, USA) for analysis. In this analysis, we compared the cases and controls for the proportions with HIV/ART status, vitamin A deficiency, cigarette smoking and for levels of UV radiation exposure. We also compared cases and controls for the mean or median for CD4 count, number of cigarettes smoked daily and serum retinol levels. CD4 counts were graded by the WHO criteria 29. All analyses were adjusted for age, sex and study centre. Multivariable logistic regression analysis was used to estimate odds ratios (ORs) and 95% confidence intervals (CIs). The likelihood ratio test was used to assess statistical significance of associations. Variables that were associated with the outcome on the initial adjusted analyses at a level of *P* < 0.05 were included in the multivariable analysis, and then those with *P* < 0.2 were retained in the model.

## Results

Five potential cases and six potential controls declined to participate. 262 participants were recruited: 131 cases and 131 controls were frequency‐matched by age group and sex. About two‐thirds were females (72% of cases and 65% of controls, *P* = 0.23). The mean age and standard deviation (SD) of cases and controls were 42.1 years (SD = 13.1) and 43.3 years (SD = 13.2), respectively (*P* = 0.43). The controls all had surgery for conditions without primary conjunctival pathology: lacerations (*n* = 41), traumatic cataract (*n* = 39), vitreous haemorrhage (*n* = 29), retinal detachment (*n* = 18) and squint surgery (*n* = 4).

After adjusting for the matching variables, OSSN was strongly associated with HIV infection (OR = 18.96, 95% CI: 8.87–40.5), lower CD4 count (OR = 37.33, 95% CI: 7.98–174.5 for CD4 < 200 cells/mm^3^ compared with CD4 > 500 cells/mm^3^) and history of allergic conjunctivitis (OR = 30.78, 95% CI: 4.05–234). There were weaker associations with lower education level (OR = 1.82, 95% CI: 1.03–3.21 for no education or primary *vs*. completed secondary or more) and outdoor occupations (OR = 1.73, 95% CI: 1.03–2.91; Table 2). Residential area was not associated with OSSN status, but there was some evidence of a higher odds of OSSN among those who resided in Nyanza Province (OR = 3.00, 95% CI: 1.04–8.65 compared with Nairobi). There was no evidence that current active smokers were at higher risk than non‐smokers (OR = 1.62, 95% CI: 0.56–4.75) – the *P* value (0.03) was influenced by a lower risk in passive smokers (OR = 0.54, 95% CI: 0.15–1.94). OSSN was also not associated with the duration of smoking (*P* = 0.79) or the number of cigarettes smoked daily (*P* = 0.60). Cases had lower median levels of serum retinol than controls (44.9 μg/dl *vs*. 51.0 μg/dl, *P* = 0.03).

**Table 2:** Analysis of potential risk factors for ocular surface squamous neoplasia adjusted for age group, sex and study centre

Exposure	Cases (*N* = 131)	Controls (*N* = 131)	OR (95% CI)	*P* value
Marital status, No. (%)				0.37
Married	83 (63.4)	89 (67.9)	1 [Reference]	
Single	22 (16.8)	26 (19.9)	0.91 (0.48–1.73)	
Widowed	18 (13.7)	10 (7.6)	1.93 (0.84–4.45)	
Divorced or separated	8 (6.1)	6 (4.6)	1.43 (0.47–4.31)	
Highest education level, no. (%)				0.04
Completed secondary or more	46 (35.1)	68 (51.9)	1 [Reference]	
Completed primary or some secondary	53 (40.5)	39 (29.8)	1.82 (1.03–3.21)	
None or some primary	32 (24.4)	24 (18.3)	1.78 (0.90–3.52)	
Residential area (province)				0.21
Nairobi	28 (21.4)	42 (32.1)	1 [Reference]	
Rift Valley	36 (27.5)	28 (21.4)	1.93 (0.96–3.88)	
Central	27 (20.6)	34 (26.0)	1.19 (0.59–2.40)	
Eastern	13 (9.9)	9 (6.9)	2.17 (0.81–5.85)	
Nyanza	14 (10.7)	7 (5.3)	3.00 (1.04–8.65)	
Western	10 (7.6)	10 (7.6)	1.50 (0.55–4.11)	
Coast	1 (0.8)	1 (0.8)	1.50 (0.09–25.52)	
North‐eastern	2 (1.5)	0	–	
Duration of residence in home province, median (IQR), y	24 (10–39)	28 (14–40)	–	0.10^a^
History of allergic conjunctivitis^b^	28 (21.5)	1 (0.9)	30.78 (4.05–233.75)	<0.001
Location of current occupation, no. (%)^b^				0.02
Indoor	45 (34.9)	62 (48.1)	1 [Reference]	
Outdoor	84 (65.1)	67 (51.9)	1.73 (1.03–2.91)	
Duration in current occupation, median (IQR), y	10 (5–20)	15 (7–23)	–	0.04^a^
Sun exposure per day in current occupation, mean (SD), h	6.9 (3.8)	4.6 (3.2)	1.22 (1.12–1.32)	<0.001^c^
Hat or cap worn outdoors, no. (%)^b^	19 (15.0)	32 (24.8)	0.60 (0.30–1.20)	0.07
Sunglasses worn outdoors, no. (%)^b^	10 (7.9)	16 (12.4)	0.66 (0.28–1.55)	0.11
Cigarette smoking (past), no. (%)^b^				0.10
No	93 (71.5)	90 (70.3)	1 [Reference]	
Passive (spouse/partner smokes)	17 (13.1)	21 (16.4)	1.09 (0.47–2.53)	
Yes	20 (15.4)	17 (13.3)	1.11 (0.54–2.31)	
Cigarette smoking (current), no. (%)^b^				0.03
No	115 (89.2)	111 (88.1)	1 [Reference]	
Passive (spouse/partner smokes)	4 (3.1)	9 (7.1)	0.54 (0.15–1.94)	
Yes	10 (7.8)	6 (4.8)	1.62 (0.56–4.75)	
Duration of cigarette smoking, mean (SD), y	13.5 (8.6)	12.7 (10.0)	–	0.79^c^
No. of cigarettes smoked daily, median (IQR), y	9 (5–20)	7 (5–10)	–	0.60^a^
HIV infection^d^, no. (%)	62 (74.7)	14 (13.6)	18.96 (8.87–40.51)	<0.001
HIV infection and ART use^e^, no. (%)				<0.001
HIV−	21 (16.0)	89 (67.9)	1 [Reference]	
HIV+/ART−	17 (13.0)	2 (1.5)	38.54 (8.19–181.39)	
HIV+/ART+	40 (30.5)	12 (9.2)	14.30 (6.35–32.20)	
No HIV or ART data	53 (40.5)	28 (21.4)	7.18 (3.67–14.05)	
CD4 count^f^, median (IQR), cells/mm^3^	251 (118–670)	796 (619–1063)	–	<0.001^a^
CD4 WHO category, no.(%), cells/mm^3^				<0.001
≥500	20 (15.3)	64 (48.9)	1 [Reference]	
350–499	6 (4.6)	9 (6.9)	2.39 (0.74–7.68)	
200–349	11 (8.4)	1 (0.8)	39.24 (4.68–328.33)	
<200	22 (16.8)	2 (1.5)	37.33 (7.98–174.50)	
No CD4 data	72 (55.0)	55 (42.0)	3.84 (2.06–7.17)	
Vitamin A deficiency (<30 μg/dl)^g^, no. (%)	5 (8.8)	5 (5.8)	1.30 (0.32–5.32)	0.50
Serum retinol, median (IQR), μg/dl	44.9 (37.6–56.2)	51.0 (39.7–64.7)	–	0.03^a^

SD, standard deviation; IQR, interquartile range.

a Wilcoxon–Mann–Whitney *U‐*test.

b There were missing data on allergic conjunctivitis (24 participants), occupational location 4, hat or sunglasses use 6 and cigarette smoking 7.

c *t*‐test.

d The denominator here is 83 cases and 103 controls who were tested for HIV.

e We used the combined HIV/ART variable because the effect of HIV would be related to ART use.

f There were 59 cases and 76 controls who had a CD4 test.

g The denominator here is 57 cases and 86 controls who had a vitamin A test.

Independent risk factors for OSSN are shown in Table 3. Participants who were HIV positive and not on ART were at the greatest risk (adjusted OR = 48.42, 95% CI: 7.73–303.3 compared with HIV‐negative participants), and there was also a very strong association with history of allergic conjunctivitis (adjusted OR = 74.61, 95% CI: 8.08–688.9). The duration of exposure to the sun per day in their occupations was higher among cases than controls (6.9 h/day *vs*. 4.6 h/day, *P* < 0.001). There was also a strong independent protective effect of wearing a hat or cap outdoors (OR = 0.22, 95% CI: 0.07–0.63).

**Table 3:** Multivariable logistic regression analysis of factors associated with OSSN

Exposure	Adj OR^a^ (95% CI)	*P* value
HIV infection and ART use^a^		<0.001
HIV−	1 [Reference]	
HIV+/ART−	48.42 (7.73–303.31)	
HIV+/ART+	19.16 (6.60–55.57)	
No HIV or ART data	9.38 (3.78–23.29)	
History of allergic conjunctivitis	74.61 (8.08–688.91)	<0.001
Sun exposure in current occupation	1.24 (1.10–1.40)	0.001
Hat or cap worn outdoors	0.22 (0.07–0.63)	0.01
Highest education level	1.55 (0.91–2.65)	0.11

The mean (SD) CD4 count with respect to HIV and ART was; HIV‐ was 926(298) cells/mm^3^; HIV+/ART‐ was 258(196) cells/mm^3^; HIV+/ART+ was 314(225) cells/mm^3^; and missing HIV/ART data was 136 (101) cells/mm^3^.

a We used the combined HIV/ART variable because the effect of HIV would be related to ART use.

## Discussion

OSSN appears to be a disease driven by multiple risk factors. In this study, we set out to examine several of these in a large case–control study in Kenya nested within a randomised controlled trial, and confirmed established risk factors such as exposure to ultraviolet radiation and HIV infection and some potential new ones such as allergic conjunctivitis.

This study design has several strengths compared with previous case–control studies. Most notably, previous studies have used controls with benign lesions which could be on the causal pathway for OSSN or could share similar risk factors, and this is likely to underestimate the magnitude of their effects. For example, HIV is an established risk factor for OSSN, but we found a much stronger association of HIV and OSSN than previously reported in a meta‐analysis of six case–control studies in East and Southern Africa (summary OR = 6.17; 95% CI: 4.83–7.89) 2. Five of those studies were conducted in the era before ART programmes were available or scaled up in the region while one was conducted in 2010 when ART was available in Ugandan public hospitals and did not report on ART use 30. HIV is also associated with more severe OSSN disease. A study in India that compared HIV‐infected OSSN patients with HIV‐negative OSSN patients found that HIV infection was associated with larger tumours and a higher incidence of extension to the cornea, sclera and orbit, but there were no data reporting ART use 31. Although we had CD4 data on only 59 cases and 76 controls, it was intriguing that CD4 count was not a significant risk factor after multivariable adjustment. There may have been confounding with ART as those with low CD4 counts would be started on treatment and their CD4 count would subsequently rise. We observed that those on ART had higher mean CD4 counts than those who were not (see footnote Table 3). There was also an overlap in the confidence intervals between HIV‐infected people on ART and not on ART suggesting that ART did not seem to significantly change the effect of HIV infection on OSSN. This is further supported by the observation that OSSN is still common in Africa despite availability of ART programmes for over a decade. Perhaps, the effect of HIV on OSSN may not be through CD4 counts but rather a yet‐to‐be‐understood pathway. HIV‐associated premature ageing could also play a role in the aetiology as advancing age increases cancer risk 32.

Another aspect of our study design is that we enrolled controls after cases had been recruited. There may be systematic differences between incident and prevalent OSSN cases which are difficult to identify in previous case–control studies that enrolled cases and controls concurrently.

Our study focused on modifiable risk factors to identify strategies for prevention. We found a similar effect for duration of solar ultraviolet exposure outdoors to a study in Uganda (adjusted OR = 1.7; 95% CI: 1.2–2.4 for cases exposed to 2–4 h and OR = 1.8; 95% CI: 1.1–3.1 for those exposed to ≥5 h daily) 11. The association between OSSN and UV radiation has been shown by results from cancer registries and the previously mentioned case–control study in Uganda 2, 11, 33. The latter had two types of controls per case: patients attending the eye clinic for reasons other than OSSN, and patients originally recruited as cases but whose histology was non‐OSSN 11. UV radiation affects the nasal limbus more than other parts of the eye which explains why there is a higher proportion of both benign and malignant conjunctival lesions nasally than temporally 4, 34, 35. Although cases had worked for a shorter period at their current occupations (10 years *vs*. 15 years, *P* = 0.04), they were more likely to have outdoor occupations than controls (65% *vs*. 52%) and had longer daily exposure to the sun at work (*P* < 0.001). The association we observed between less education and OSSN could have been due to those less educated people (particularly incomplete secondary school level) having jobs with greater sun exposure (means range from 6.5 to 8.4 h/day) than controls (4.6 h/day), but it persisted on adjustment suggesting residual confounding or an independent effect. The observed protective effect of wearing hats while outdoors is consistent with UV radiation being a risk factor of OSSN. The earlier Uganda study did not find hats protective (OR = 1.3; 95% CI: 0.8–2.2) 11. We did not find sunglasses protective similar to the same Uganda study.

We found a relatively high prevalence of history of allergic conjunctivitis among cases (21.5%) which contrasts with previous studies. The aforementioned case–control study in Uganda reported no OSSN cases with allergic conjunctivitis, and a descriptive study in Tanzania found a prevalence of 1.9% 11, 36. The Uganda study examined for allergy in the nasal quadrant of the conjunctiva, but they were unlikely to find allergy in adults in their 30s and 40s as typically the onset is in childhood and is rare after the age of 30 years. Allergic conjunctivitis is common in Africa. Studies in Ghana and Nigeria found allergic conjunctivitis in up to 40% of school children and 33% of university students, respectively 37, 38. The disease also tends to be quite severe with limbal hypertrophy as shown in a Rwandan study where 98% of school children with vernal keratoconjunctivitis (VKC) had limbal disease 39. Although not fully understood, limbal stem cells may be the progenitors in the pathophysiology of OSSN 40. It is unclear whether allergic conjunctivitis or its treatment with steroids is the cause per se. One hypothesis may be that chronic allergic conjunctivitis is associated with a milieu of inflammation mediators which increases the probability of DNA errors during mitosis of stem cells at the limbus. Using corneal confocal microscopy, a study found that patients with VKC had more cells with a high nuclear‐to‐cytoplasm ratio (26.4% *vs*. 7.5%), more activated cells (59% *vs*. 10%) and inflammatory cells (75.4% *vs*. 0) than controls 41. Another possible explanation is that prolonged steroid use causes immunosuppression (like HIV might) which predisposes to OSSN.

There was little evidence of an association with smoking in this study, and prevalence of smoking was low. Two studies in Uganda showed evidence of no effect of current smoking compared to non‐smokers 11, 42. The levels of serum retinol were lower in cases suggesting that vitamin A may play a role. This is biologically plausible as previous studies have demonstrated ocular surface changes related to vitamin A deficiency such as keratinisation which presents as leukoplakia, a frequent feature of OSSN lesions 21. Squamous metaplasia of the ocular surface epithelium is also seen in retinol deficiency and reversed by topical retinoid therapy 43. Retinoic acid, a metabolite of retinol, has antitumour effects and helps to maintain the pluripotency of limbal stem cells through specific receptors in the cell nucleus 44.

This study had several limitations. Selection bias may be present if potential controls with a less serious disease than OSSN were not inclined to participate. The refusal rates were however not different for cases at 5 of 273 (1.8%) and 6 of 273 (2.2%) for controls. Recall bias could not be ruled out as cases are more likely to remember about exposures more than controls. We had considerable missing data on HIV, CD4 count and vitamin A as not all excised cases returned post‐operatively for testing. The classification of occupations is not ideal. We would have preferred one that classifies occupations by possible exposure to carcinogens. Existing systems such as Kenya National Occupational Classification System (KNOCS) which is derived from the International Standard Classification of Occupations (ISCO) are designed for only economic purposes and not for epidemiological studies 45.

In conclusion, we identified modifiable risk factors for OSSN. HIV control interventions are now available, exposure to UV radiation can be reduced, and indeed we found that wearing hats was protective, while allergic conjunctivitis can be better managed in younger people. The results of this study corroborate what is known about the pathophysiology of OSSN where the limbus stem cells appear to be the main focus of disease. However, the latency period between first exposure to these risk factors and causation of OSSN is still not known. The role of CD4 count in OSSN remains to be elucidated, and why OSSN usually affects one eye is still an enigma. Measures to control HIV, prevent sun exposure such as wearing hats and control allergic conjunctivitis are recommended.

## Supporting information


**Table S1.** Analysis of factors that may potentially cause bias in the comparison of cases of ocular surface squamous neoplasia and controls adjusted for age group, sex and study centre.

**Table S2**. The histological grade and TNM stage of the cases.
