# Supplementary material for: Risk factors for ocular surface squamous neoplasia in Kenya: a case–control study
Source: Trop Med Int Health. 2016 Oct 24;21(12):1522–30. doi: 10.1111/tmi.12792 (PMC5574019; doi:10.1111/tmi.12792)
Supplement: Supplementary file 1 — Table S1. Analysis of factors that may potentially cause bias in the comparison of cases of ocular surface squamous neoplasia and controls adjusted for age group, sex and study centre. [file TMI-21-1522-s001.docx]

Supplementary table 1. Analysis of factors that may potentially cause bias in the comparison of cases of ocular surface squamous neoplasia with controls adjusted for age group, sex and study centre.

| **Variable** | **Cases**  **(N=131)** | **Controls**  **(N=131)** | **OR (95% CI)** | ***P* value** |
| --- | --- | --- | --- | --- |
| Recruitment centre, n(%) |  |  |  | 0.05 |
| Kikuyu Eye Unit | 79 (60.3) | 99 (75.6) | 1 [Reference] |  |
| Kenyatta National Hospital | 8 (6.1) | 6 (4.6) | 1.67 (0.55 - 5.04) |  |
| Sabatia Eye Hospital | 24 (18.3) | 11 (8.4) | 2.73 (1.24 - 6.01) |  |
| Kitale District Hospital | 20 (15.3) | 15 (11.5) | 1.67 (0.80 - 3.49) |  |
| Distance from residence to study center, median(IQR), km | 32 (15 - 96) | 43 (27- 125) | - | 0.002^a^ |
| Tribal group, n(%) |  |  |  | 0.36 |
| Bantu | 90 (68.7) | 100 (76.3) | 1 [Reference] |  |
| Nilote | 39 (29.8) | 30 (22.9) | 1.44 (0.83 - 2.52) |  |
| Cushite | 2 (1.5) | 1 (0.8) | 2.22 (0.20 - 25.1) |  |

Abbreviations: SD, standard deviation; IQR, interquartile range

^a^ Wilcoxon-Mann-Whitney U test
