# Supplementary material for: Risk factors for ocular surface squamous neoplasia in Kenya: a case–control study
Source: Trop Med Int Health. 2016 Oct 24;21(12):1522–30. doi: 10.1111/tmi.12792 (PMC5574019; doi:10.1111/tmi.12792)
Supplement: Supplementary file 2 — Table S2. The histological grade and TNM stage of the cases. [file TMI-21-1522-s002.docx]

Supplementary table 2. Histological grade and TNM stage of the cases.

| **Grade or stage** | **n (%)** |
| --- | --- |
| Histological grade, n(%) |  |
| CIN 1 | 7 (5.6) |
| CIN 2 | 17 (13.0) |
| CIN 3 | 34 (25.9) |
| Carcinoma-in-situ | 1 (0.9) |
| Well differentiated squamous cell carcinoma | 2 (1.9) |
| Moderately differentiated squamous cell carcinoma | 51 (38.9) |
| Poorly differentiated squamous cell carcinoma | 18 (13.9) |
| Total | 131 (100.0) |
|  |  |
| TNM stage ^a^ |  |
| T1N0M0 | 27 (20.4) |
| T2N0M0 | 15 (11.1) |
| T3N0M0 | 75 (57.4) |
| T3N0M1 | 5 (3.7) |
| T3N1M0 | 4 (2.8) |
| T3N1M1 | 1 (0.9) |
| T4N0M0 | 1 (0.9) |
| T4N0M1 | 2 (2.9) |
| T4N1M1 | 1 (0.9) |
| Total | 131 (100.0) |

Abbreviations: TNM – tumour, node, metastasis

^a^ After the American Joint Classification of Cancer (AJCC) staging system
